# HIV Infection, Antiretroviral Therapy and Cardiovascular Risk

**DOI:** 10.4084/MJHID.2010.034

**Published:** 2010-11-11

**Authors:** Katleen de Gaetano Donati, Roberto Cauda, Licia Iacoviello

**Affiliations:** 1 Department of Infectious Diseases, Catholic University Medical School, Largo A. Gemelli 8, 00168 Roma, Italy; 2 Laboratory of Genetic and Environmental Epidemiology, ”John Paul II” Center for High Technology Research and Education in Biomedical Sciences, Catholic University, Largo A. Gemelli 1, 86100 Campobasso, Italy

## Abstract

In the last 15 years, highly active antiretroviral therapy (HAART) has determined a dramatic reduction of both morbidity and mortality in human immunodeficiency virus (HIV)-infected subjects, transforming this infection in a chronic and manageable disease. Patients surviving with HIV in the developed world, in larger number men, are becoming aged. As it would be expected for a population of comparable age, many HIV-infected individuals report a family history of cardiovascular disease, a small proportion have already experienced a cardiovascular event and an increasing proportion has diabetes mellitus. Smoking rate is very high while an increasing proportion of HIV-infected individuals have dyslipidaemia. Studies suggest that these traditional risk factors could play an important role in the development of cardiovascular disease in these patients as they do in the general population. Thus, whilst the predicted 10-year cardiovascular disease risk remains relatively low at present, it will likely increase in relation to the progressive aging of this patient population. Thus, the long-term follow-up of HIV infected patients has to include co-morbidity management such as cardiovascular disease prevention and treatment. Two intriguing aspects related to the cardiovascular risk in patients with HIV infection are the matter of current investigation: 1) while these subjects share many cardiovascular risk factors with the general population, HIV infection itself increases cardiovascular risk; 2) some HAART regimens too influence atherosclerotic profile, partly due to lipid changes. Although the mechanisms involved in the development of cardiovascular complications in HIV-infected patients remain to be fully elucidated, treatment guidelines recommending interventions to prevent cardiovascular disease in these individuals are already available; however, their application is still limited.

## Introtuction:

The advent of highly active antiretroviral therapy (HAART), providing sustained suppression of viral replication and preservation of immune system function, has made human immunodeficiency virus (HIV) infection a chronic and manageable disease for many patients.^1^ The increased life expectancy and the effects of HAART have changed the management of HIV infection: medical treatment is no longer limited to HIV infection but also includes the control of metabolic, cardiovascular, liver, bone and kidney complications.

**Figure 1.**
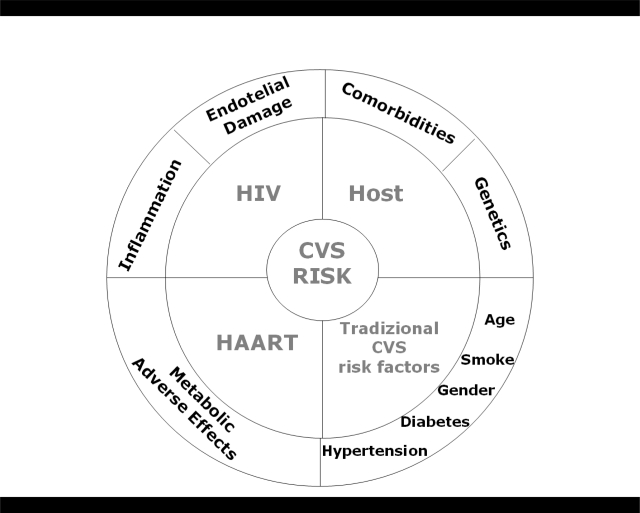
Cardiovascular risk in HIV-positive subjects can be attributed to host genetics, traditional risk factors, adverse effects from antiretroviral therapy or the inflammatory state associated with HIV itself.

In particular, understanding the risk of cardiovascular disease (CVD) in persons with HIV infection is complex. Controversies exist as to how much such risk can be attributed to host genetics, traditional risk factors, adverse effects from antiretroviral therapy or the inflammatory state associated with HIV itself.^2^ Thus, all factors potentially contributing to CVD need to be considered when managing persons infected with HIV (Figure 1).

**Figure 2.**
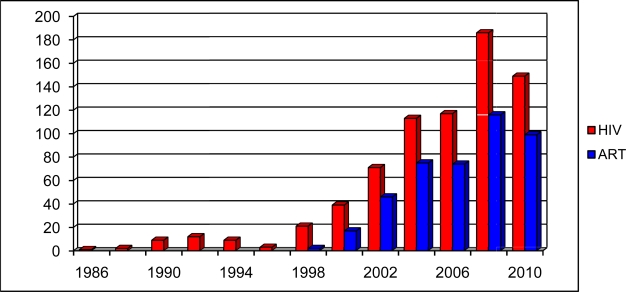
“Cardiovascular risk factors” AND “HIV” OR “antiretroviral therapy” citations in PubMED from 1986 until July 2010.

In the last 25 years, a deep and increasing effort to study HIV infection has been performed by a multidisciplinary approach: infectious disease physicians, epidemiologists, cardiologists and oncologists participated to this work with different expertises and expectations. Cross-checking in Pubmed the words “cardiovascular risk” and “hiv” or “cardiovascular risk” and “antiretroviral therapy” for each single year from 1985 until now, it is clear that there has been an increasing interest at first in the relationship between HIV infection itself and cardiovascular risk and then, from 1996 - when HAART became a standard for HIV-positive patients- between antiretroviral therapy and cardiovascular risk (Figure 2). It is just shortly after the introduction of HAART, that case reports of myocardial infarction (MI) and early vascular atherosclerotic lesions in young patients infected with HIV were published.^3^ Although the initial focus was primarily on the protease inhibitors (PI), suggesting that lipodystrophy and its related metabolic disorders could increase cardiovascular risk,^4^ a broader appreciation of the complex interplay between traditional risk factors for CVD and HIV infection has emerged more recently.

Several groups of investigators have designed studies to examine various aspects of the relationship between HIV infection, traditional cardiovascular risk factors, HAART, and short- and longer-term cardiovascular risk.^5^–^10^ Studies have included both clinical end points (MI, hospitalization for MI or angina, and revascularization) and surrogate markers of atherosclerosis (endothelial function or carotid intima-media thickness). Further studies have included data on traditional risk factors, longer follow-up, and diverse patient populations.

## Methodological Limitations:

The difficulties in determining risks among patients with HIV infection have largely been due to the lack of matched controls, small sample size, and lack of standardized definitions, besides the unknown contribution from HIV itself. Often, data sources developed for other purposes have been used for a better understanding of the association between HIV and CVD. These studies vary in end points, methods of end-point collection and validation, degree to which data on traditional risk factors were captured, and the amount of information available about the type and duration of HAART exposure. When HIV-negative control groups were included, they were often not matched for important, traditional risk factors that may be more prevalent in HIV groups, such as smoking. Studies including HIV and non HIV-infected control subjects should indeed contain complete information on traditional risk factors, including smoking, to assess the relative contribution of these risk factors to CVD rates and to determine whether HIV *per se* is a marker for patients with increased traditional CVD risk markers. Despite methodological limitations inherent to the use of pre-existing or administrative databases, some consistent research themes have emerged in this area, namely, that traditional CVD risk factors and relative CVD disease rates are increased in HIV-infected patients, although absolute rates remain low in the HIV population.

## Global Cardiovascular Risk in HIV-Infected Subjects:

Unfortunately, the principal studies in this area are not reliable from a cardiology point of view: a recent attempt by our group to perform a meta-analysis confirmed the great confusion existing on this topic (Table 1). From this point of view, every attempt to perform a meta-analysis will not be useful until controlled clinical trials will be performed with a tight collaborations between cardiologists and infectivologists, with a careful monitoring and treatment of all cardiovascular risk factors and with a careful definition and analysis of cardiovascular events. Too much confounding factors do not allow to properly analyze the weight of antiretroviral therapy compared to other defined cardiovascular risk factors in the development of cardiovascular events.

However, certain studies have shown that there may be increased risk of CVD in HIV-infected versus uninfected populations (Table 2). Discordant results have been however obtained, which possibly reflect differences in end-point definitions and ascertainment or differences in underlying cardiovascular risk in the various populations studied. Klein and collaborators^10^ provided one of the first reports comparing rates of hospitalization for CVD in HIV-infected adults with that of uninfected control subjects. These Authors have demonstrated that HIV patients have a higher risk of hospitalizations for CVD and specifically for acute MI relative to HIV-uninfected controls. Currier et al^5^ observed an increased incidence of CVD in younger HIV-infected men and women as compared to HIV- uninfected recipients. One more recent study also reported an excess relative risk of CVD among HIV-infected adults compared with HIV-uninfected control subjects. Triant et al.^8^ in fact, compared MI rates among HIV-infected adults and found higher rates of acute MIs in HIV-infected individuals than in HIV-uninfected adults. They also noted a higher prevalence of conventional risk factors for CVD, such as hypertension, diabetes mellitus, and dyslipidemia, in the HIV-positive group, factors that separately and especially in combination could explain, at least in part, the excessive risk of CVD in the HIV-infected group.

## Traditional Cardiovascular Risk Factors:

It is critical to understand whether increased CVD rates are causally linked to HIV-related factors or merely reflect differences in the prevalence of underlying traditional risk factors. Importantly, studies that have controlled for these factors have consistently shown a significant effect of traditional risk factors on CVD events in HIV-infected patients.^7^–^8^ Age, smoking, hypertension and diabetes mellitus are all strong predictors of CVD risk in HIV-infected patients. Additionally, the background prevalence rates of these factors in many HIV cohorts are high and may, for some, predict the acquisition of HIV infection. In particular, rates of smoking in HIV populations are consistently high and exceed those for age-matched controls in several studies.^17^–^18^

According to the results of DAD (the largest multinational cohort collecting data on adverse effects of anti-HIV drugs), the risk associated with protease inhibitor was considerably lower than the annual increase in risk associated with advanced age, male sex or current smoking.^6^ In fact, the cross-sectional analysis on risk factors of the same cohort showed that among 17,852 HIV-positive patients 25% presented an increased cardiovascular risk for age, 51% for smoking, and there was a high prevalence of known cardiovascular risk factors.^17^

### Age:

The proportion of HIV-infected patients older than 50 years has greatly increased since the beginning of the epidemic. By 2015, 50% of HIV-infected individuals in the United States are expected to be older than 50 years.^19^ The incidence and prevalence of HIV infection in older adults is rising, with disproportionate increase in women and minorities. Compared with younger adults, older patients who have HIV are often diagnosed later and may have an accelerated decline in immune function.^20^ Although the prognosis for older adults has improved with the initiation of HAART,^21^ a higher risk for co-morbid illness remains.^22^

Rather than occurring merely as a consequence of extended survival among ART recipients, the increased rate of CVD may result from accelerate biological aging imposed by HIV itself and/or antiretroviral treatments. Coronary artery calcium (CAC) has been used to describe the biological age of an individual, which may be different from his chronological age. The use of CAC provides an objective tool to assess premature biological aging in HIV-infected patients.^23^ Additionally, a risk score that includes CAC and age may be an easily understandable measure of risk.^24^ On the other hand, asymptomatic, HIV-infected men with long-lasting HIV disease have an increased prevalence and degree of coronary atherosclerosis compared with non-HIV-infected patients.^25^ In treated patients who achieve durable suppression of the HIV virus, natural ageing, drug specific toxicity, lifestyle factors, persistent inflammation, and perhaps residual immunodeficiency are all causally associated with premature development of complications normally associated with ageing, including cardiovascular disease.^26^

### Smoking:

Smoking rates range from 35% to 72% in clinical studies of HIV-positive subjects.^18^ Over 85% of HIV-infected individuals in the U.S. have a lifetime history of smoking and current cigarette smoking is highly prevalent among HIV-positive persons. On average, HIV-positive smokers have been smoking for 22.8 years and smoke 16 to 23 cigarettes per day. Among HIV-positive current smokers, most are moderately to highly nicotine dependent.^27^

### Diabetes:

Although guidelines in the general population consider diabetes mellitus (DM) to be equivalent to coronary heart disease (CHD), there is little information on its association with CHD in subjects infected with HIV. DM and preexisting CHD are both important risk factors for CHD events in HIV-infected individuals. There is a need for targeted interventions to reduce the risk of CHD in both high-risk groups of HIV-infected individuals.^28^

### Dyslipidemia:

There is substantial evidence that HIV infection impacts on blood lipids, but the interplay between infection, treatment and changes in various lipid parameters is complex.^29^,^30^ Initiation of multidrug antiretroviral therapy usually results in increased lipids. Protease inhibitors tend to induce greater increase in total cholesterol, low-density lipoprotein cholesterol (LDL-c) and triglyceride than non-nucleoside reverse transcriptase inhibitors (NNRTI) whereas nevirapine has been linked to increase in high-density lipoprotein (HDL-c). Protease inhibitor–induced cholesterol changes at least partly explain the increased CHD risk observed in treated compared with untreated HIV-infected people.^31^

The prevalence of dyslipidemia, whether genetically determined or influenced by HAART (elevated triglycerides, total cholesterol, and LDL-c) or HIV infection (low HDL-cholesterol), is consistently higher in HIV groups.

### Metabolic Syndrome:

A number of changes seen with HIV infection, restoration to health, and treatment with HAART, including dyslipidemia, diabetes, increased body mass index and waist circumference, may present simultaneously in HIV-infected patients.

These factors are part of the metabolic syndrome. It is debated whether the prevalence of the metabolic syndrome is increased among HIV-infected patients. Our group has observed that HIV-positive patients over 45 years presented a prevalence of metabolic syndrome of 49% versus 31% among controls, taken from the Moli-sani cohort^32^ of HIV-negative subjects matched for sex, cardiovascular risk factors and cancer (p<0.01) (data not published).

It remains unknown whether the presence of the metabolic syndrome *per se* confers increased risk for CVD disease in HIV-infected patients beyond that associated with individual risk factors. Furthermore, metabolic syndrome has several features in common with the lipodystrophy syndrome observed in HIV–positive patients, such as insulin resistance, dyslipidaemia and fat redistribution.^33^–^35^

The relative contribution of each of the cardiovascular risk factors depicted is similar in HIV-infected and uninfected populations, which suggests that these factors contribute to cardiovascular risk in a comparable way irrespective of their HIV status. Consistent with this, analyses that compare the observed incidence of CHD in HIV-infected populations with that predicted from risk equations developed in the general population have reported reasonably similar outcomes. Hence, traditional cardiovascular risk factors contribute in important ways to the risk of CVD in HIV.

## Short and Long-Term Antiretroviral Therapy:

### ...From Bozzette to SMART Through DAD..

Bozzette and his group^9^ reported that overall mortality due to HIV dramatically declined in the early HAART era without any increase in admissions for cardiovascular or cerebrovascular events. Although limited by the short duration of exposure to combination HAART and by possibly incomplete capture of events, this study suggested that over the short term, the benefits of HAART clearly outweighed the risk for CVD. The same Authors, more recently, confirmed similar conclusions regarding global reduction of mortality and hospitalization for CVD in patients with exposure to all antiretroviral drug classes.^36^

Recently, several well-designed prospective studies have shed some light on the complex interactions between the use of antiretroviral therapy, HIV infection and cardiovascular risk. The Strategies for Management of Antiretroviral Therapy (SMART) study^37^ reported that interruption of antiretroviral therapy was associated with an increased risk of opportunistic disease or death. Furthermore, the drug-conservation strategy in the SMART trial was associated with a 60% increase in the risk of CVD during a mean follow-up of 16 months only, indicating that effective viral suppression actually may reduce short-term cardiovascular risk.^37^ The implication of this study is that short-term use of antiretroviral therapy reduces cardiovascular risk. However, the long-term effects of such therapy on cardiovascular disease are unclear; in this context the first report of the DAD study group is interesting: among 23,437 patients who were followed for a median of 4.5 years, there were 345 myocardial infarction with an incidence of 3,7/1000/year. Among these events 29% were fatal representing 10% of all the study deaths.^6^ Thus increased exposure to PI was associated with an increased risk of MI, a finding that was partly explained by dyslipidemia. Thus short or long-term risks of antiretroviral therapy may differ.

The second DAD report, confirmed the result of the first one: MI incidence rate increased in relation to increasing antiretroviral therapy exposure with a relative risk of 1,16/year of exposure. No data are available regarding other longer term associations. It is interesting that the association between antiretroviral therapy exposure and cardiovascular risk remains after adjusting for age and sex. After adjusting for cardiovascular risk factors, except lipids, patients with PI showed a 16% increase risk of MI per year versus 5% per year among patients with NNRTI. Further adjustment for lipids reduced the increased risk to 10% for PI and eliminated the association with NNRTI use^7^ (Table 3).

It is critical to recognize that the magnitude of increased cardiovascular risk observed with PI is not high, especially as compared with the effect of other cardiovascular risk factors. The relative risk per year of exposure to PI was 1.16, which is considerably smaller than the relative risk of increasing age, male sex, current smoking or history of cardiovascular diseases.

Recently, the DAD study unexpectedly found almost a double rate for MI in HIV-infected patients treated with *abacavir*, a nucleoside reverse trascriptase inhibitor (NRTI), for the previous 6 months. The risk associated with abacavir use was independent of traditional cardiovascular risk factors and was no longer significant when abacavir had been stopped prior to the last 6 months, suggesting an ‘on–off ’ mechanism directly induced by abacavir that might involve biological mechanisms associated with atherosclerosis.^40^ A *posthoc* analysis of the SMART study also showed a higher risk of cardiovascular disease, including MI, in patients treated with abacavir.^41^ These patients had higher levels of inflammation and hypercoagulability biomarkers (interleukin-6, IL-6 and high sensitivity C reactive protein, hs-CRP) at the SMART study entry. Abacavir could have induced those pathogenetic mechanisms, owing proinflammatory properties, that induce flogosis of arterial wall and subsequent instability of already present plaques. In this way expression of a subclinical atherosclerosis could become clinically apparent. By contrast, data from naive patients in a Glaxo-Smith-Kline (GSK) and Aids Clinical Trial Group (ACTG) studies did not show any significant difference regarding MI between abacavir-treated and un-treated antiretroviral naive patients.^42^,^43^ The potential cardiovascular effects of abacavir might be less evident in antiretroviral-naive patients because of the confounding noise due to uncontrolled HIV replication. Two major sources of bias and confounding not controlled for in previous studies, such as drug prescription and uncontrolled HIV infection, did not affect a more recent study,^44^ that provided an opportunity to assess the potential effects of abacavir on different mechanisms involved in the pathogenesis of MI as compared to other antiretroviral drugs. Such study showed that abacavir/lamivudine increased both total and LDL cholesterol compared with tenofovir/emtricitabine, but did not cause any inflammation, endothelial dysfunction, insulin resistance, or hypercoagulability in virologically suppressed HIV-infected patients.

In a recent analysis of DAD data, Worm and co-workers^45^ examined the risk of MI associated with exposure to individual antiretroviral drugs from three major drug classes: the individual drugs examined, namely indinavir, lopinavir-ritonavir, abacavir, and didanosine were all associated with an increased risk of MI.

This risk appeared to increase with cumulative exposure to the two PIs and could partly be explained by dyslipidemia caused by these drugs. In contrast, associations between MI risk and abacavir and didanosine exposure were largely confined to those patients with recent exposure to the drugs and did not appear to be driven by dyslipidemia. As the overall rate of MI remains relatively low, any toxicities of antiretroviral drugs must always be balanced *versus* the benefits that these drugs provide. In agreement with these results, Jong and colleagues^46^ recently showed that HIV-infected patients using abacavir had no specific abnormalities in coagulation or inflammation markers that might explain the increased risk of MI. For the whole group, regardless of abacavir use, evidence of a pro-thrombotic state was observed. Thirty-three percent of patients with long-term use of antiretroviral treatment had hs-CRP levels above 3 mg/L, which is strongly associated with cardiovascular disease in HIV-uninfected individuals.^46^

## Inflammation and Endothelial Function:

The ACTG 5152 Study^47^ had the purpose of evaluating the effects of three HAART regimens on endothelial function in treatment-naïve HIV-infected subjects participating in a large, prospective, randomized trial. Endothelial function was evaluated by measuring flow-mediated dilation (FMD) of the brachial artery by B-mode ultrasound. Patients with HIV infection who met criteria for starting HAART had impaired endothelial function. Use of three different ART regimens rapidly improved endothelial function in treatment-naive patients with HIV infection. Improvement was similar for all HAART regimens, appeared quickly, and persisted over 24 weeks. It is unclear if the improvements were due to HAART, suppression of viremia, or changes in factors not measured in this study, such as immune activation or biological variation.^47^

A review by our group^48^ compared different studies on endothelial adhesion molecule levels looking for their increase or reduction during HAART. If soluble adhesion molecules (and other endothelial cell molecules) are reliable biomarkers of pathological endothelial activation by HIV, the obvious corollary would be that a beneficial treatment should lower their levels in the circulation. Endothelial dysfunction, as measured by circulating cell adhesion molecule concentrations, should thus be reduced by HAART: while HIV infection impact generally increases the endothelial adhesion molecule levels, HAART impact, except for some studies, generally reduces such levels, suggesting that therapy reduces indeed endothelial damage.

In apparent contrast with this last assumption and previous reports, we had obtained data supporting the hypothesis that HAART does indeed induce activation of endothelial function. In particular, we measured intercellular adhesion molecule-1 (ICAM-1) and P-selectin concentrations (as well as tissue plasminogen activator, tPA and plasminogen activator inhibitor-1, PAI-1), in a group of HIV-positive patients undergoing either PI or NNRTI therapy and compared them with naïve HIV-positive patients. P-selectin, t-PA, and PAI-1 levels were all significantly higher in both HAART subgroups, while ICAM-1 concentrations did not differ significantly from those measured in the naive group. In addition, a positive association was seen between lipid levels and endothelial biomarkers—namely, cholesterol correlated with t-PA, P-selectin, and ICAM-1, while triglycerides correlated with PAI-1. We reasoned that, on one hand—by controlling HIV infection—HAART should reduce the associated endothelial injury; on the other hand—by deranging lipid metabolism—it would contribute to stimulating endothelial function.^49^

Thus a substantial proportion of the risk attributed to PI remains unexplained. Although the DAD study demonstrates a relative increase in risk with increased duration of HAART, in part due to the presence of one or more traditional risk factors, the absolute risk of CVD will remain low for most patients. Because the absolute CVD rates remain low, the relative increase in these rates might not have public health significance: however this situation may change in the future as HIV patients will live longer due to successful HAART.^50^

C reactive protein (CRP) is an indicator of immune activation in response to inflammatory damage or infection and has been shown to increase in HIV-1–infected individuals. Levels of CRP were associated with HIV disease progression independent of CD4 lymphocyte counts and HIV RNA levels. In addition, regardless of progression to AIDS, HIV-infected individuals had a significant increase in CRP over time. Fibrinogen and CRP are strong and independent predictors of mortality in HIV-infected adults. Even in those with relatively preserved CD4 counts >500 cells per microliter, inflammation remains an important risk factor for mortality.^51^,^52^

The higher prevalence of premature carotid lesions in the PI-treated patients requires the routine introduction of a periodic ultrasonographic study of the vascular wall in the follow-up of HIV infected patients.^53^

The presence of subclinical carotid lesions has been shown to highly associate with the estimated Framingham Risk Score (p<0.002). The presence of subclinical atheromasic lesions was also high among antiretroviral-naïve patients. HIV infection per se is a risk factor for atherosclerosis. Thus an ultrasonographic assessment both among patients with FRS 6% or more and among those in advanced stage of disease is required.^54^

## Viro-Immunological Features:

The DAD study^7^ found no association between either the peak HIV-1 RNA level or the nadir CD4+ lymphocyte count and the risk of MI, although the possibility that other unmeasured immunologic effects could have exerted an influence on the development of CVD cannot be excluded. The Staccato trial^55^ in fact, investigated whether HIV replication modified the levels of plasma soluble inflammatory molecules in a combination antiretroviral therapy interruption trial. Initiation of HAART resulted in significant declines in s-VCAM-1, P-selectin, leptin and D-dimer, whereas mediators with anti-inflammatory properties, such as adiponectin and IL-10, increased. At 12 weeks after randomization, they found positive associations between levels of s-VCAM-1 and chemokine ligand 2 with an increase in plasma HIV-RNA, whereas levels of adiponectin decreased for each 1 log increase in plasma HIVRNA. Plasma levels of several inflammatory, anti-inflammatory and endothelial activation markers of cardiovascular disease are associated with HIV-RNA replication.^55^ On the other hand, Kaplan and colleagues^56^ observed that beyond traditional cardiovascular disease risk factors, low CD4+ T-cell count is the most robust risk factor for increased subclinical carotid atherosclerosis in HIV-infected women and men.

## Co-morbidities:

Several studies demonstrated that the presence of co-morbidities, such as viral hepatitis, renal and bone diseases, could influence cardiovascular risk in HIV-positive patients.^57^–^58^

## Cardiovascular Risk Evaluation:

Although the estimation of CVD risk might prove useful both to establish the relative contribution of different risk factors and for clinical management in HIV patients, estimates of CVD risk from long term observational studies are still scanty in these settings, and estimates of CVD using algorithms other than Framingham are lacking.^59^ De Socio and colleagues^60^ have recently used Framingham Risk Score as well as three other cardiovascular algorithms (“PROCAM”, “PROGETTO CUORE”, “SCORE”) to calculate the 10-year probability of acute coronary events in HIV-positive patients. The utilization of these tools to identify patients with a worse CV risk profile seems to be a promising and simple way for monitoring long term CVD risks in the population of HIV infected patients, as cardiovascular risk reduction efforts will necessarily rely on combined interventions, given the multiplicity of risk factors involved.

## Cardiovascular Risk Management:

*“Choosing an effective antiretroviral regimen has become an art”* writes Aberg, considering the difficulties to choose a regimen that allow to obtain viral suppression without any adverse effect of HAART such as increased cardiovascular risk.^61^

Cardiovascular risk should be evaluated before initiation of antiretroviral therapy and frequently thereafter during follow-up, and decisions to alter therapy on the basis of adverse changes in metabolic risk factors should be made on an individual basis. Virologic control is the primary goal for HIV-infected persons with cardiovascular risk,^62^ and is the primary consideration in determining when to start antiretroviral therapy and to change regimens. Current guidelines suggest treating cardiovascular risk in HIV-infected patients in the same manner as recommended for the general population. Management may include dietary and exercise intervention, smoking cessation, establishment of lipid goals and treatment of dyslipidemia, while drug therapy (eg, statins, antihypertensives, aspirin) should only be added in high-risk patients (eg, those with established coronary disease, diabetes, or moderate or high risk on risk scoring). Switching of antiretroviral therapy may be considered, when other methods of treating risk are not effective.

Collectively, the data linking viremia and endothelial dysfunction and inflammation, the increased risk of cardiovascular events with treatment interruption, and the association between cardiovascular disease and CD4 cell depletion, suggest that early control of HIV replication with antiretroviral therapy can be used as a strategy to reduce cardiovascular disease risk.^63^

## Conclusions:

As in the general population, individual cardiovascular risk factors such as hypertension, diabetes, dyslipidemia, and smoking have an additive or synergistic impact on overall risk and should be addressed at initiation of antiretroviral therapy and frequently during follow-up. Lifestyle modification should be the first management approach, including smoking cessation, diet modification, and increased exercise. In managing hyperlipidemia, the decision to use lipid-lowering therapy or to switch antiretroviral therapy regimens should be individualized. The impact of smoking cessation is greater than the impact of any other intervention in reducing overall risk, and although cardiovascular risk should be considered when starting or changing antiretroviral therapy, virologic control should be the overriding consideration.

## Figures and Tables

**Table 1.** Attempt of a meta-analysis of clinical cardiovascular events in HIV-positive subjects: 11 populations including 145,448 HIV-positive patients presented 4,207 clinical events.

**Studies [references]**	**Year**	**N° Patients**	**Type of event**	**N° Events**
Rickerts V. et al. [11]	2000	4,993	AMI	29
Klein D. et al. [10]	2002	4,159	CAD/AMI	72
Holmberg S.D. et al [12]	2002	5,672	AMI/CVD	35
Currier J.S. et al. [5)	2003	28,513	CAD/AMI	1,360
Bozzette S.A. et al. [9]	2003	36,766	CAD/CVD	2,006
Murielle M.K. et al. [13]	2003	34,976	AMI	60
Barbaro G. et al. [14]	2003	1,551	AMI	26
Varriale P. et al. [15]	2003	690	AMI	29
Escaut L. et al. [16]	2003	840	CAD/AMI	17
Triant V.A. et al. [8]	2007	3,851	AMI	189
DAD [7]	2007	23,437	IMA/CVD	384

AMI=acute myocardial infarction, CVD=cerebrovascular diseases, CAD=coronary artery disease

**Table 2.** Global cardiovascular risk in HIV-infected patients compared with HIV-negative subjects

**References [n°]**	**Event rate per 1,000 HIV + patients**	**Event rate per 1,000 HIV − patients**
Klein [10]	4.3	2.9
Currier [5]	4.1	3.3
Triant [8]	11.1	6.9

**Table 3.** Association between cardiovascular risk and HAART: it exists?

**Retrospective studies [references]**	**Association between cardiovascular risk and HAART**
Klein D. et al. [10]	No
Bozzette S.A. et al. [9]	No
Mary-Krause M. et al [4]	Yes
Currier J.S. et al. [5]	Yes
Obel N. et al. [38]	yes
Vaughn G. et al [39]	yes
**Prospective studies (references)**	
Holmberg S.D. et al [12]	yes
DAD [7]	yes
**Randomized clinical trial (references)**	
SMART [37]	no
